# Relationship between recurrence and age in the diffuse sclerosing variant of papillary thyroid carcinoma: clinical significance in pediatric patients

**DOI:** 10.3389/fendo.2024.1359875

**Published:** 2024-06-20

**Authors:** Junu Kim, Ja Seung Koo, Ji-In Bang, Jin Kyong Kim, Sang-Wook Kang, Jong Ju Jeong, Kee-Hyun Nam, Woong Youn Chung

**Affiliations:** ^1^ Department of Surgery, Severance Hospital, Yonsei Cancer Center, Yonsei University College of Medicine, Seoul, Republic of Korea; ^2^ Department of Pathology, Severance Hospital, Yonsei Cancer Center, Yonsei University College of Medicine, Seoul, Republic of Korea; ^3^ Department of Nuclear Medicine, CHA Bundang Medical Center, CHA University, Seongnam, Gyeonggi-do, Republic of Korea

**Keywords:** thyroid neoplasm, thyroid cancer, papillary thyroid cancer, pediatrics, diffuse sclerosing variant, prognosis

## Abstract

**Background:**

The diffuse sclerosing variant (DSV) is among the aggressive variants of papillary thyroid carcinoma (PTC) and is more prevalent in pediatric patients than in adult patients. Few studies have assessed its characteristics owing to its low incidence. We aimed to evaluate the relationship between recurrence and age in the DSV of PTC.

**Methods:**

We retrospectively reviewed patients diagnosed with the DSV or conventional PTC (cPTC) after surgery at a medical center between May 1988 and January 2019. We compared the clinico-pathological characteristics and surgical outcomes of the DSV and cPTC groups and between adult and pediatric patients with DSV.

**Results:**

Among the 24,626 patients, 202 had the DSV, and 24,424 were diagnosed with cPTC. The recurrence rate was significantly higher in the DSV group than in the cPTC group. In the DSV group, the recurrence rate was significantly higher in the pediatric patient group than in the adult patient group. Moreover, the association between recurrence and age group showed different patterns between the DSV and cPTC groups with restricted cubic splines (RCS). While both RCS curves showed a U-shaped distribution, the RCS curve tended to be located within the younger age group.

**Conclusions:**

This study demonstrated that pediatric patients with DSV are at a greater risk for recurrence compared with adult patients; moreover, the pattern of recurrence risk according to age is different from that of cPTC.

## 1 Introduction

Papillary thyroid carcinoma (PTC) has an indolent behavior and a favorable prognosis (1, 2). However, 10–20% of PTC cases show aggressive features, resulting in frequent recurrence and sometimes higher mortality (3–5). The prognostication of DTC remains significantly constrained by low specificity (6), despite advancements in understanding the prognostic implications of tumor-specific genetic alterations (7). From the perspective of the prognostic field of thyroid cancer, there is a pressing need for prognosticators with high specificity and positive predictive value regarding the rate of recurrent disease after initial treatment.

The rare diffuse sclerosing variant (DSV) is among the representative aggressive subtypes of thyroid cancer that tends to occur in young patients (8–10), with a varying reported prevalence of 0.8–5.3% of PTC cases (11–16). DSV was first described in 1985 (17) and is characterized by aggressive clinico-pathological manifestations, including diffuse involvement of one or both thyroid lobes without a dominant mass, sclerosis, abundant psammoma bodies, prominent squamous metaplasia, extensive lymphatic permeation, and stromal fibrosis (18, 19). The outcomes and prognosis of DSV remain controversial (9, 10, 12–15, 20–22); however, in cases where preoperative sonography images show typical features of DSV, such as ill-defined margins, scattered microcalcifications with a snowstorm appearance, and varying echogenicity (23), radical treatment is pursued more frequently. This is due to the aggressive and highly recurrent nature of DSV, which distinguishes it from cases without these characteristics (24, 25).

To date, most studies have focused on the aggressiveness of the DSV by comparing it with conventional PTC (cPTC) (9, 18, 20–22). However, the recurrence pattern of the DSV itself remains unknown. Moreover, the relationship between recurrence and age in patients with DSV has not yet been investigated, although DSV is a prevalent and major variant of pediatric PTC (8).

To the best of our knowledge, this is the first study to perform a subgroup analysis of the DSV. In addition to confirming the well-known characteristics of the DSV compared with those of cPTC, we compared the characteristics of pediatric and adult patients with the DSV. In particular, we focused on the associations between age and recurrence rates in patients with DSV.

## 2 Materials and methods

### 2.1 Patient identification and data collection

We retrospectively analyzed the data of patients who were diagnosed with DSV or cPTC after undergoing surgery at a medical center in South Korea between May 1988 and January 2019. Demographic characteristics, including tumor histology, age, sex, and pathology results, were collected from electronic medical records. TNM staging was performed according to the American Joint Committee on Cancer 8^th^ edition guidelines (25). Recognizing the varying definitions of pediatric age across countries and institutions (26), we defined the pediatric age as individuals under the age of 20 years based on the definition used in a previous publication from the same institution (8). All surgeries, including those involving pediatric patients, were performed by specialized thyroid and endocrine surgeons within the General Surgery Department’s Thyroid and Endocrine Surgery Division. All patients underwent at least central compartment dissection, either therapeutically or prophylactically (27). Patients who had additional cancers, distant metastases, a history of thyroid surgery, or underwent follow-up for less than 3 years at our institution after surgery were excluded from the study. All patients received annual neck sonography for approximately 5 years from the date of surgery; after this, they underwent neck sonography at least once every 2 years. A chest CT was performed at least once within 5 years after surgery.

For each DSV case, the mutational status of the *BRAF 600E* or *TERT* genes was evaluated if the test results existed. In our institution, *BRAF 600E* and *TERT* mutational analyses have been performed routinely since 2014 and 2019, respectively. This study was conducted in accordance with the tenets of the Declaration of Helsinki (as revised in 2013) and approved by the institutional review board (IRB) of Yonsei University (IRB number: 4–2020-1257). The requirement for informed consent was waived owing to the retrospective nature of this study.

### 2.2 Definition of recurrence

Recurrence was defined as structural recurrence, including local recurrence or distant metastases, which required active surgical or medical intervention. Biochemical evidence of disease without structural evidence was not considered as recurrence in this study. Recurrence-free survival (RFS) was counted from the operation date until the date of pathological confirmation of loco-regional recurrence or the date of the imaging study that provided definite evidence of distal metastases.

In cases where locoregional recurrence was suspected, fine-needle aspiration biopsy was performed, and wash-thyroglobulin levels were checked. For cases with suspected distant metastasis, treatments such as RAI ablation were actively applied. There were no cases in which the patients were solely placed under surveillance without intervention.

### 2.3 Statistical analysis

Statistical analyses were performed using IBM SPSS Statistics for Windows software (version 26.0; IBM, Armonk, NY). First, we determined the clinical, pathological, and treatment-related characteristics of the DSV and cPTC groups, as well as those of adult and pediatric patients. Categorical data were compared between groups using the chi-squared test; continuous numerical data with normal distribution are described as means ± standard deviations and were compared between groups using the Student’s t-test. Non-normally distributed data were compared using the Wilcoxon rank sum test. RFS was examined using Kaplan–Meier survival curves. Univariate and multivariate Cox proportional hazards regression were used to identify factors associated with recurrence. Restricted cubic splines (RCS) were used to analyze the relationship between age and RFS (28, 29). RCS analysis provides a visual model with which to examine the relationship between a continuous parameter (age) and an outcome (recurrence) using interpolation and smoothing of data points while adjusting for other factors (tumor size). The RCS model (knot number, 3) was used to estimate hazard ratios (HRs) with 95% confidence intervals (CIs) at different ages in reference to the age with the lowest HR. The number of knots was determined according to previous studies (28, 29), and knots were placed at the 10^th^, 50^th^, and 90^th^ percentiles of patient age. RCS models were constructed using the mgCV and rms packages in R (version 3.2.4; R Foundation for Statistical Computing, Vienna, Austria). Statistical significance was set at p <0.05.

## 3 Results

### 3.1 Characteristics of patients with cPTC compared with patients with DSV

We analyzed data from 24,626 patients diagnosed with cPTC or the DSV between May 1988 and January 2019. Among them, 24,424 (99.2%) were diagnosed with cPTC, whereas 202 (0.8%) were diagnosed with the DSV. The total follow-up period was 97.9 ± 52.5 months.

The clinico-pathological characteristics of the DSV and cPTC groups were compared (**Table 1**). The proportion of males was significantly higher in the DSV group than in the cPTC group (29.48% vs. 20.50%, respectively; p=0.038). Patients in the DSV group were significantly younger than those in the cPTC group (mean age: 32.9 ± 12.7 years vs. 45.0 ± 11.8 years, respectively; p<0.001). There was an over-representation of younger patients in the DSV group (17.8%) compared with the cPTC group (0.6%) (p<0.001).

**Table 1:** Clinicopathologic features of patients with conventional papillary thyroid carcinoma vs. patients with the diffuse sclerosing variant cPTC vs. DSV variant.

Variable	cPTC (24, 424)	DSV (202)	p-value
**Sex (F:M, M(%))**	20,268:4,156 (20.50%)	156:46 (29.48%)	0.038
**Age, years**	45.0 ± 11.8	32.9 ± 12.7	<0.001
Age group, years			<0.001
**- <20**	148 (0.6%)	17.8%)	
**- 20 ≤ age <55**	18,907 (77.4%)	5.7%)	
**- ≥ 55**	5,369 (22.0%)	13 (6.4%)	
**Tumor size, cm**	0.9 ± 0.7	1.8 ± 1.1	<0.001
**Capsule invasion, n (%)**	12,632 (51.7%)	155 (76.7%)	<0.001
**Multifocality, n (%)**	2,560 (10.5%)	21 (10.4%)	<0.001
**Bilaterality, n (%)**	4,326 (17.7%)	99 (49.0%)	<0.001
Central node, n			
**- Total**	5.5 ± 4.7	11.1 ± 7.0	<0.001
**- Metastatic**	1.1 ± 2.2	7.0 ± 5.6	<0.001
**- Lymph node ratio (metastatic/total)**	0.2 ± 0.3	0.6 ± 0.3	<0.001
Lateral node, n			
**- Total**	33.7 ± 16.6	47.2 ± 25.2	<0.001
**- Metastatic**	5.6 ± 5.0	10.4 ± 6.7	<0.001
**- Lymph node ratio (metastatic/total)**	0.18 ± 0.23	0.23 ± 0.13	<0.001
Operative extent			<0.001
**- Lobectomy**	8,155 (33.4%)	19 (9.4%)	<0.001
**- Lobectomy with contralateral partial**	3,800 (15.6%)	6 (3.0%)	
**-Total thyroidectomy**	12,469 (51.1%)	177 (87.6%)	
Neck lymph node dissection			<0.001
**None**	385 (1.6%)	1 (0.5%)	
**CCND**	21,753 (89.1%)	68 (33.7%)	
**MRND**	2,286 (9.4%)	133 (65.8%)	
**RAI-treated**	10,555 (43.2%)	168 (83.2%)	<0.001
T stage			<0.001
**T1**	1,880 (77.3%)	116 (57.4%)	
**T2**	611 (2.5%)	37 (18.3%)	
**T3**	4,397 (18.0%)	32 (15.8%)	
**T4**	536 (2.2%)	17 (8.4%)	
N stage			<0.001
**N0**	14,914 (61.1%)	21 (10.4%)	
**N1a**	7,277 (29.8%)	48 (23.8%)	
**N1b**	2,233 (9.1%)	133 (65.8%)	
TNM stage 8^th^			<0.001
**Stage I**	20,884 (85.5%)	190 (94.1%)	
**Stage II**	942 (3.9%)	10 (10.0%)	
**Stage III**	2,112 (8.6%)	2 (2.0%)	
**Stage IV**	486 (2.0%)	0 (0%)	
**No. of recurrences**	600 (2.5%)	19 (9.4%)	<0.001
**No. of disease-specific deaths**	76 (0.3%)	0 (0.0%)	

cPTC, conventional papillary thyroid carcinoma; DSV, diffuse sclerosing variant; CCND, central compartment neck dissection; MRND, modified radical neck dissection.

**Figure 1:**
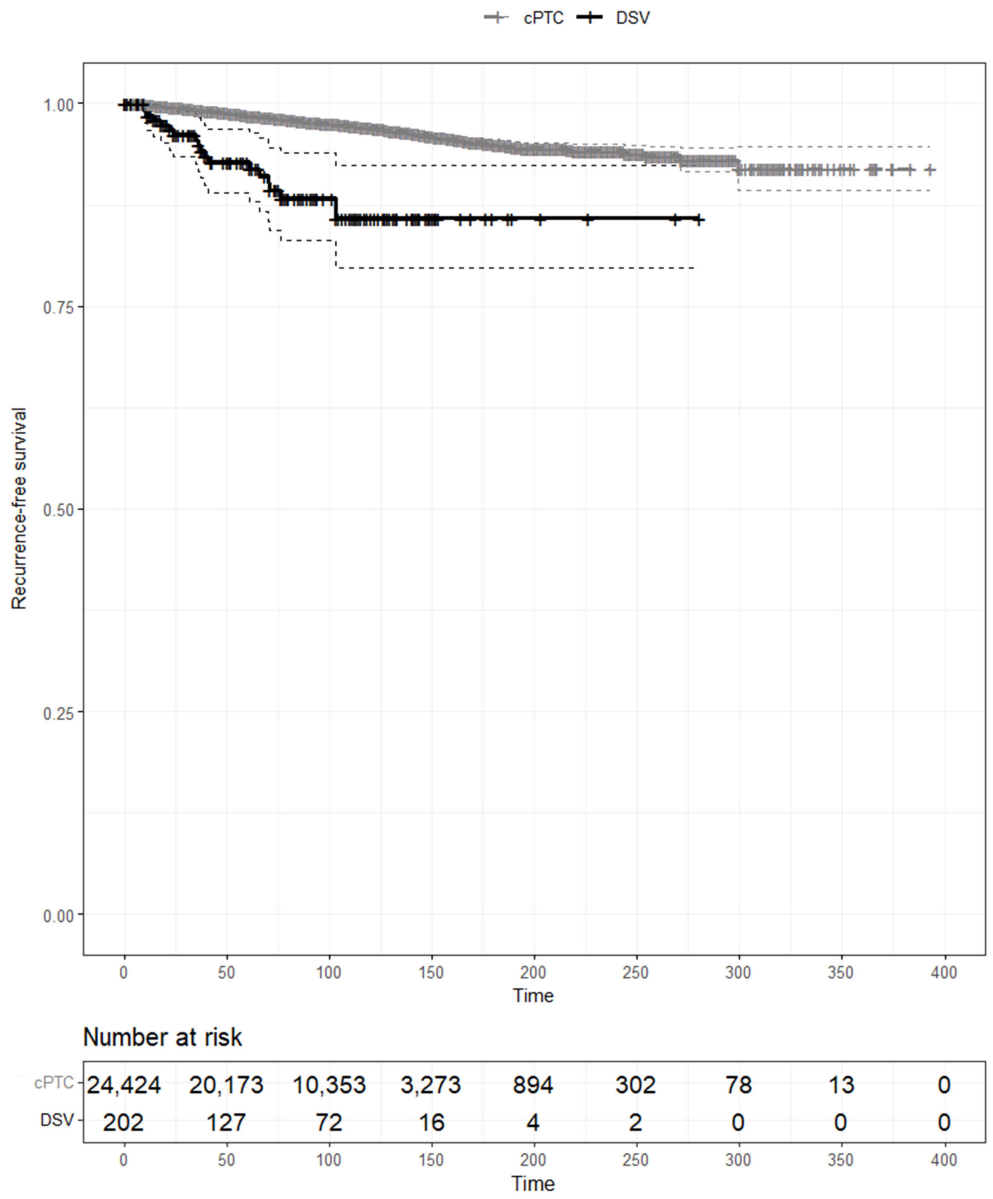
Kaplan–Meier analysis of recurrence-free survival curves of patients with conventional papillary thyroid carcinoma and those with the diffuse sclerosing variant.

The DSV group also presented with more aggressive pathological characteristics compared with the cPTC group, including a larger tumor size, more extensive tumor capsular invasion, lymph node metastasis, a higher lymph node ratio of both central cervical and lateral neck nodes, and a more advanced T and N stages, although the TNM stage was lower (all p<0.001). Accordingly, more radical treatments were performed in patients with the DSV than in patients with cPTC, including total thyroidectomy (87.6% vs. 51.1%, respectively), modified radical neck dissection (65.8% vs. 9.4%, respectively), and RAI treatment (83.2% vs. 43.2%, respectively). The recurrence rate was significantly higher in the DSV group (9.4%) than in the cPTC group (2.5%) (p<0.001). In contrast, the disease-specific mortality rate in the DSV group was 0.0% compared with 0.3% in the cPTC group. In the Kaplan–Meier estimates of RFS, the DSV was significantly associated with a worse RFS compared with cPTC (p<0.01) (**Figure 1**).

### 3.2 Comparison of the characteristics of pediatric and adult patients in the DSV group

**Figure 2:**
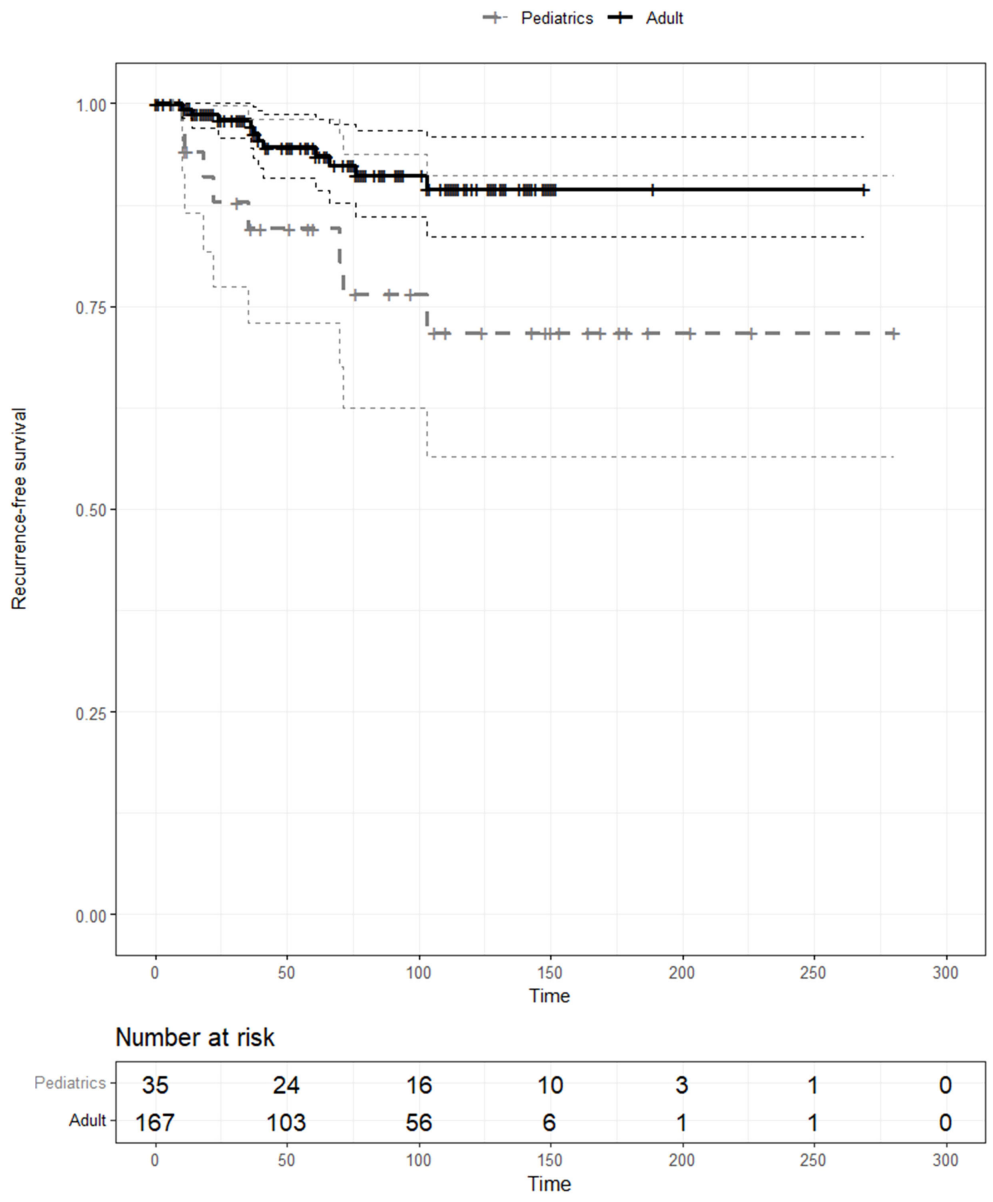
Kaplan–Meier analysis of recurrence-free survival curves of pediatric and adult patients with the diffuse sclerosing variant.

The demographic and clinico-pathologic characteristics of pediatric (n=35) and adult (n=167) patients with DSV were compared (**Table 2**). The two groups did not differ significantly in terms of sex, the presence of tumor capsular invasion, multifocality and bilaterality, node conditions, or operative extent (p<0.05). In contrast, the tumor size and T stage were significantly higher in the pediatric population (p<0.001 and p=0.001, respectively). The recurrence rate was also significantly higher in pediatric patients (22.9%) than in adult patients (6.6%) (p=0.007). The Kaplan–Meier estimates for RFS were also compared between pediatric and adult patients with the DSV, and RFS was significantly lower in pediatric patients with the DSV compared with adult patients with cPTC (p<0.01) (**Figure 2**).

**Table 2:** Comparison of pediatric vs. adult patient characteristics.

Variable	Pediatric (N=35)	Adult (N=167)	p-value
**Sex (F:M, F(%))**	29:6	127:40	0.515
**Age, years**	15.2 ± 3.3	36.4 ± 10.4	<0.001
**Tumor size, cm**	2.5 ± 1.4	1.6 ± 1.0	<0.001
**Capsule invasion, n (%)**	28 (80.0%)	127 (76.0%)	0.777
**Multifocality, n (%)**	3 (8.6%)	18 (10.8%)	0.583
**Bilaterality, n (%)**	16 (45.7%)	83 (49.7%)	0.556
Central node, n			
**- Total**	11.0 ± 6.6	11.1 ± 7.1	0.862
**- Metastatic**	7.1 ± 5.4	7.0 ± 5.7	0.813
**- Ratio**	0.6 ± 0.3	0.6 ± 0.3	0.612
Operative extent			0.085
**- Lobectomy**	6 (17.1%)	13 (7.8%)	
**- Lobectomy with contralateral partial**	2 (5.7%)	4 (2.4%)	
**- Total thyroidectomy**	27 (77.1%)	150 (89.8%)	
Neck lymph node dissection			0.160
**- None**	1 (2.9%)	0 (0.0%)	
**- CCND**	13 (37.1%)	55 (32.9%)	
**- MRND + CCND**	21 (60.0%)	112 (67.1%)	
**RAI-treated**	26 (74.3%)	142 (85.0%)	0.122
T stage			0.001
**- T1**	11(31.4%)	106 (63.5%)	
**- T2**	12 (34.3%)	23 (13.8%)	
**- T3**	8 (22.9%)	26 (15.6%)	
**- T4**	4 (11.4%)	12 (7.2%)	
N stage			0.686
**- N0**	5 (14.3%)	16 (6.6%)	
**- N1a**	8 (22.9%)	40 (24.0%)	
**- N1b**	22 (62.9%)	111 (66.5%)	
TNM stage 8^th^			0.456
**- Stage I**	35 (100%)	155 (93.4%)	
**- Stage II**	0	10 (6.0%)	
**- Stage III**	0	2 (1.2%)	
**- Stage IV**	0	0	
**No. of recurrences**	8 (22.9%)	11 (6.6%)	0.007

DSV, diffuse sclerosing variant; CCND, central compartment neck dissection; MRND, modified radical neck dissection; CI, confidence interval; HR, hazard ratio.

CCND, central compartment neck dissection; MRND, modified radical neck dissection.

### 3.3 Mutational status of patients with DSV

In this study, *BRAF* and *TERT* mutation results were only available for 65 and 21 patients, respectively. Among them, 22 patients (33.8%) were *BRAF* mutation-positive, and only 1 patient (4.7%) was *TERT* mutation-positive.

### 3.4 Risk analysis of recurrence of the DSV

In total, 19 recurrence events were described in the DSV group. The demographic and clinico-pathological factors of interest described above were entered into a Cox proportional-hazards regression model with the recurrence rate as the dependent outcome (**Table 3**). The prognostic factors associated with a higher recurrence rate at the univariate level were belonging to the pediatric patient group vs. adult patient group (HR=3.17; 95% CI: 1.27–7.89; p=0.013), larger tumor size (HR=1.34; 95% CI: 1.01–1.77; p=0.042), and non-referral for total thyroidectomy (HR=0.32; 95% CI: 0.11–0.97; p=0.045). In the multivariate analysis, belonging to the pediatric patient group compared with the adult patient group was the only significant predictor of recurrence (HR=2.82; 95% CI: 1.11–7.16; p=0.047) after adjusting for tumor size.

**Table 3:** Comparison of Cox regression models between the adult and pediatric groups.

Variables	Univariate analysis			Multivariate analysis		
	HR	95% CI	p-value	HR	95% CI	p-value
Female vs. Male						
** Female **						
** Male**	2.12	0.83–5.38	0.096			
Pediatric vs. Adult						
** Adult**						
** Pediatric**	3.17	1.27–7.89	0.013	2.82	1.11–7.16	0.047
**Age**	0.97	0.93–1.01	0.094			
**Tumor size**	1.34	1.01–1.77	0.042	1.22	0.90–1.66	0.194
**Capsule invasion (+)**	6.79	0.91–50.91	0.063			
**Central node ratio**	1.18	0.29–4.85	0.822			
**Lateral node ratio**	3.94	0.05–301.62	0.540			
Operative method						
**- Lobectomy**						
**-Lobectomy with contralateral partial**	0.59	0.07–5.24	0.631			
**- Total thyroidectomy**	0.32	0.11–0.97	0.045			
RAI						
**None ** ** Treated**	0.474	0.22-2.02	0.668			
Stage T						
**T1**						
**T2**	1.55	0.41–5.84	0.519			
**T3**	2.21	0.72–6.78	0.166			
**T4**	3.09	0.82–11.65	0.097			
Stage N						
**N0**						
**N1a**	1.34	0.27–6.63	0.722			
**N1b**	1.07	0.24–4.83	0.931			

**Figure 3:**
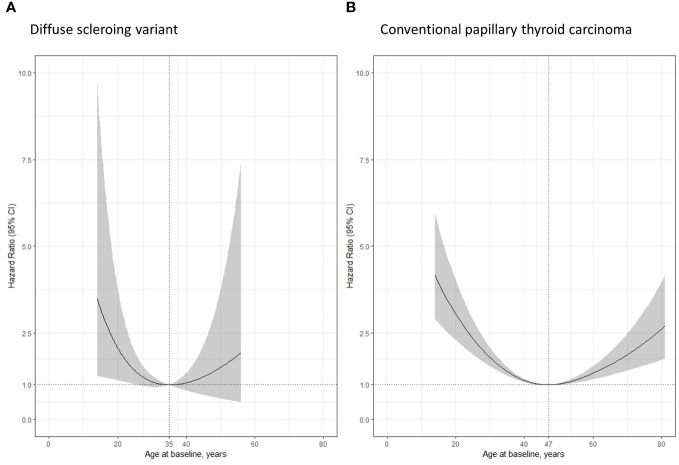
Restricted cubic spline (RCS) according to patients’ age, adjusted for tumor size. **(A)** Patients with the diffuse sclerosing variant showed the lowest hazard ratio of recurrence at the age of 35 years; **(B)** Patients with conventional papillary thyroid carcinoma showed the lowest hazard ratio of recurrence at the age of 47 years.

Lastly, we used RCSs, which are more adaptive, smoother splines, to assess the relationship between age and the risk of DSV- or cPTC-specific recurrence (**Figure 3**). The RCS curves of both groups showed a U-shaped distribution, and the lowest HR of each group was deliberately set at 1. The lowest HR occurred at 35 years of age in the DSV group and at 47 years of age in cPTC group. The RCS curve of the DSV tended to be located within the younger age group.

## 4 Discussion

To the best of our knowledge, this is the first study to perform a subgroup analysis of the DSV. Treating patients with DSV is often a sobering experience for clinicians owing to the aggressive pattern of the disease and its high recurrence rate. Moreover, knowledge regarding which patient characteristics (including age, sex, maximal tumor diameter, lymph node condition, and TNM stage) should be monitored more carefully for the DSV remains largely unknown.

In the current staging system of differentiated thyroid cancer, older age is a well-established risk factor for mortality (30). Contrarily, it is also well-known that the recurrence pattern of PTC is not exactly correlated with the staging system. For instance, although pediatric patients with PTC have a relatively high recurrence rate, they also have an excellent prognosis in terms of mortality (31). Considering the low mortality rate of thyroid cancer, it is important to investigate its recurrence pattern, which is directly linked to the patients’ quality of life, requiring additional active treatment such as re-operation or repetitive radioiodine ablation, especially for aggressive subtypes such as the DSV.

In this study, our data shared similar results with previous studies. First, the DSV accounted for 0.8% of PTC cases, which is similar to the results of previous studies (11–16). Second, the DSV had a higher incidence in pediatric patients than in cPTC patients (8). Third, patients with the DSV had larger tumor sizes and lymph node metastases compared with patients with cPTC, resulting in more aggressive treatment (18, 19). Fourth, recurrence rates were higher in patients with the DSV compared with patients with cPTC. Fifth, despite its high recurrence rate, the DSV was associated with excellent survival rates (9, 18, 21, 22). Finally, the prevalence of *BRAF or TERT* mutations was low in patients with the DSV, which is acceptable in comparison with previous results (32–34).

Although there was a significant difference in the number of patients with cPTC (n=24,424) compared with the number of patients with DSV (n=202), no statistical matching was performed as the main purpose of this study was to perform a subgroup analysis of the DSV group.

Notably, we demonstrated that pediatric status is an independent risk factor for the recurrence of the DSV. We used only two variables for the multivariate Cox proportional hazard regression analysis owing to the limited number of events. Nevertheless, we obtained an HR of 2.82 (p=0.047) for recurrence in the pediatric patient group compared with that in the adult patient group with the DSV, even when controlling for tumor size.

It is interesting that pediatric differentiated thyroid cancer (DTC) and DSV are both known to be associated with high recurrence but with excellent survival as well. They also share similar molecular characteristics of less *BRAF 600E* or *TERT* mutations. Considering the dominance of the DSV in pediatric patients with DTC (8), we hypothesized that the reason for the distinctive characteristics of DTC in pediatric patients may be the predominance of the DSV. The present study provides an important basis for future research on this topic.

The interpretation of RCS curves requires careful attention (**Figure 3**). It is noteworthy that the RCS curve of both the DSV and cPTC groups showed a U-shaped distribution, with the curve of the DSV tending to be located within the younger age group. In the DSV group, the RCS curve of the population whose age was not less than 55 years was not depicted owing to the low frequency and recurrence rates. Among the 202 patients with the DSV, only 13 were aged 55 years or above, and there was only 1 patient (a 57-year-old man) with recurrence (i.e., lung metastases within 10 months of the initial total thyroidectomy). The oldest patient in the DSV group was a 70-year-old woman who did not show any signs of recurrence within 36 months after the initial surgery. Therefore, we are still uncertain whether the prognosis of patients with DSV can be predicted using the current staging system, which dichotomizes patients’ age at 55 years across different types of thyroid cancer and considers older patients as having a worse prognosis.

To date, it remains unclear whether younger patients (<10–15 years of age) are at a greater risk of recurrence of DTC (31). In our study, the three youngest patients were aged <10 years (6, 7, and 9 years of age at diagnosis) and had no recurrence for at least 10 years after their initial diagnosis. The nine pediatric patients with recurrence were aged between 12 and 19 years, indicating a high recurrence rate among teenagers in the DSV group.

This study had several limitations. First, the study had a retrospective design. Second, we did not test for *RET/PTC* rearrangement, which is known as a major genetic alteration of the DSV. Third, all patients were treated at a single tertiary hospital, which may have caused selection bias. Fourth, we limited recurrence to structural recurrence. Clinicians often encounter cases of abnormal serum thyroglobin levels without structurally identifiable disease in clinical practice, indicating the need to be vigilant of biochemical recurrence; hence, this may have contributed to systematic bias in our study. Fifth, although we collected the data from 202 patients with the DSV, more independent risk factors can be obtained from a larger population. Variables such as tumor size, total thyroidectomy, and extracapsular invasion were only marginally insignificant in our study.

## 5 Conclusions

This study demonstrated that pediatric patients with DSV are at a greater risk for recurrence compared with adult patients; moreover, the pattern of recurrence risk according to age is different from that of cPTC.

## Data availability statement

The raw data supporting the conclusions of this article will be made available by the authors, without undue reservation.

## Ethics statement

The studies involving humans were approved by Institutional Review Board of Yonsei University. The studies were conducted in accordance with the local legislation and institutional requirements. Written informed consent for participation was not required from the participants or the participants’ legal guardians/next of kin in accordance with the national legislation and institutional requirements.

## Author contributions

JK: Writing – original draft, Resources, Investigation, Formal analysis, Data curation. JSK: Writing – review & editing, Validation, Supervision, Software, Conceptualization. J-IB: Writing – review & editing, Validation, Supervision, Software, Formal analysis, Data curation. JKK: Writing – review & editing, Writing – original draft, Visualization, Validation, Supervision, Software, Resources, Project administration, Methodology, Investigation, Data curation, Conceptualization. S-WK: Writing – review & editing, Validation, Supervision, Data curation. JJ: Writing – review & editing, Validation, Supervision, Data curation. K-HN: Writing – review & editing, Validation, Supervision, Data curation. WC: Writing – review & editing, Validation, Supervision, Data curation.
